# Mortality in children with classic congenital adrenal hyperplasia and 21-hydroxylase deficiency (CAH) in Germany

**DOI:** 10.1186/s12902-018-0263-1

**Published:** 2018-06-08

**Authors:** Helmuth G. Dörr, Hartmut A. Wollmann, Berthold P. Hauffa, Joachim Woelfle, on behalf of the German Society of Pediatric Endocrinology and Diabetology

**Affiliations:** 1 Paediatric Endocrinology, Department Paediatrics, University Hospital of Erlangen, Erlangen, Germany; 2 Department Paediatrics, University Hospital of Tübingen, Tübingen, Germany; 3 Paediatric Endocrinology, Department Paediatrics, University Hospital of Essen, Essen, Germany; 4 Paediatric Endocrinology, Department Paediatrics, University Hospital of Bonn, Bonn, Germany

**Keywords:** Adrenal crisis, CYP21A2, 21-hydroxylase deficiency, Mortality

## Abstract

**Background:**

Adrenal crises in children with classic congenital adrenal hyperplasia due to 21-hydroxylase deficiency (CAH) are life-threatening and have the potential to death.

**Methods:**

A survey was performed among Paediatric Endocrinologists in Germany to report on deceased children with CAH. Our survey covered the whole of Germany.

**Results:**

The participating centres reported 14 cases of death (9 female, 5 male) from 1973 until 2004, but no deaths thereafter. 11 children had the SW form and 3 the simple virilizing (SV) form. All patients were on glucocorticoid replacement, and the SW forms additionally on mineralocorticoid replacement. The age at death varied between 6 weeks and 16.5 years. Seven children died before introduction of general neonatal screening, and 7 children thereafter. Before death, the clinical signs of impending crisis were nonspecific. Five patients developed hypoglycaemia and convulsions with cerebral oedema. Half of the deceased patients died at home. The hydrocortisone dosage was only doubled in two of the 14 cases.

**Conclusions:**

According to the assessments by the attending centres, almost all deaths could be related to an inadequate administration of stress doses of hydrocortisone. Since no deceased CAH children were reported in Germany from 2005 on, we assume the effectiveness of educational programs over the past years.

**Electronic supplementary material:**

The online version of this article (10.1186/s12902-018-0263-1) contains supplementary material, which is available to authorized users.

## Background

Congenital adrenal hyperplasia (CAH) comprises a group of autosomal recessively inherited disorders of cortisol biosynthesis in the adrenal cortex. More than 95% of the disorders are based on a defect of CYP21A2 (21-hydroxylase). The classic defect of CYP21A2 occurs in two forms: CAH with salt wasting (SW) and simple virilizing CAH [1–4]. The reported disease incidence varies between 1:15,000 and 1:16,000 in Europe and North America and 1:19,000 in Japan [5–7]. The treatment of choice is lifelong glucocorticoid replacement and, additionally, mineralocorticoid replacement in the SW-form of the disease [8–13]. Adrenal crisis occurring within the scope of stress situations is especially feared and a major cause of morbidity and mortality in children [14]. In adults with classic CAH, the frequency of adrenal crises was 5.8 per 100 patient years [15], and Falhammer et al. reported on an increased mortality in adults with SW-CAH [16]. Reports on mortality of children with CAH vary between 3 and 13% [17–19].

The aim of our study was to collect data from deceased CAH children in Germany and to review the cases in an attempt to determine the factors that may have contributed to their death.

## Methods

Our survey covered the whole of Germany, i.e. also the situation before reunification of East and West Germany in 1990. All members of the German Society of Paediatric Endocrinology and Diabetology in Germany participated and were asked to report on deceased children with classical CAH. The first survey was conducted between September 2001 and September 2002, the second survey was conducted in 2015. Overall 88 centres including all Paediatric University Hospitals in Germany responded, and 11 centres reported 14 cases of death (9 females, 5 males). Additionally, three deaths in adults between 21 and 32 years of age were reported, but the data were not included in our analysis. General newborn screening for CAH was introduced in Germany in 1999. The reported cases of deaths occurred during the period from 1973 to 2004; 7 cases (5 females) before screening, and 7 cases (4 females) thereafter.

A questionnaire with common questions regarding clinical background data of the deceased patients (see Additional file 1) was sent to the 11 clinics, but the questionnaire was not always fully completed because the records on file in the hospitals concerning the circumstances or cause of death were incomplete.

There is no accredited registry in Germany for children with CAH. In 1997, the AQUAPE CAH initiative (AQUAPE: Arbeitsgemeinschaft für Qualitätssicherung in der pädiatrischen Endokrinologie) was installed for prospective documentation of clinical follow-up data in patients with CAH. The database does not mirror the real situation in Germany since the participation is voluntarily and therefore not all centres provide data. Cases of deceased children are not reported in this database.

Our survey was conducted upon approval of the German Society of Paediatric Endocrinology and Diabetology (DGKED). Consent was obtained from the paediatric endocrinologists that completed the questionnaire, and the data of the deceased children was collected anonymously by a questionnaire in accordance with the Declaration of Helsinki. The study was approved by the local ethics committee of the Dept. Paediatrics of Erlangen.

## Results

Of the 14 CAH patients who died, 11 patients were clinically characterized with the salt-wasting (SW) form, and three female children with the simple virilizing (SV) form. All patients were born in Germany and received regular replacement of glucocorticoids. The patients with the SW form also received mineralocorticoids (Table 1). Molecular genetic classification of the severity of CAH according to Krone et al. [20]. The genotype Group “Null” included patients with biallelic mutations that resulted in completely inactive enzymes (e.g. gene deletions), and Group “A” included patients with homozygous I2G or heterozygous I2G in trans with a null mutation. The genetic classification was only possible in 8 cases, because 6 children were not analysed. The genotype Null was found in 5 children, and genotype A in 3 children, i.e., all these children had a severe form of CAH. The number of girls with CAH who died before newborn screening was slightly higher (*n* = 5) than the number of girls (*n* = 4) who died after screening. The sex ratio (female to male) was 2.5 before screening and 1.3 after screening. Two children died at 6 weeks and 10 months of age, whereas most children died as toddlers (*n* = 9) between 1 and 5 years of age. This group also includes a boy who was in a persistent vegetative state in connection with an Addisonian crisis at the age of 1 ½ years and lived in a home for severely handicapped children for years before he died of pneumonia at the age of 13. One boy died at school age (7 years). Moreover, two adolescents died at the age of 16. Almost all deceased patients had German parents (*n* = 12); in one case, the parents were from Turkey, and in another case, the parents were not married (German mother, Turkish father). The attending resident physicians included 10 paediatricians and 4 general practitioners.

**Table 1:** Clinical data of children with CAH who died between 1973 and 2004 in Germany

Cases	CAH Form	Sex	Age at Death	Symptoms	Place of death	Comments
1	SW	F	6 wks.	fever, vomiting	at home	no GC increase
2 ^a^	SW	M	2.5 yrs.	fever, respiratory infection	at home	cerebral oedema
3	SW	M	3.5 yrs.	fever, seizure	tertiary centre	cerebral oedema
4 ^a^	SV	F	5.0 yrs.	fever, seizure	tertiary centre	cerebral oedema
5	SW	F	2.5 yrs.	fever, vomiting	at home	no GC increase
6	SV	F	16 yrs.	gastroenteritis, salmonella	at home	no GC increase
7	SW	F	1 yr.	fever, dehydration	secondary centre	no GC increase
8	SW	M	13 yrs.	fever, vomiting, seizure	secondary centre	no GC increase
9 ^a^	SW	F	10 mo.	fever, MCAD deficiency	at home	unclear?, SIDS?
10 ^a^	SW	F	3 yrs.	fever, gastroenteritis, diarrhoea	secondary centre	cerebral oedema
11	SW	M	7 yrs.	fever, seizure	tertiary centre	no GC increase cerebral oedema
12	SW	M	16.5 yrs.	fever, chicken pox	at home	no GC increase
13	SW	F	2.5 yrs.	fever, vomiting	tertiary centre	no GC increase
14	SV	F	1.5 yrs.	fever, vomiting	at home	no GC increase

Autopsy^a^; performed on 4 children

Case 8: Addisonian crisis at the age of 18 months; since that event: persistent vegetative state (children’s home)

Case 9: MCAD = Medium-Chain-Acyl-CoA-Dehydrogenase

Abbreviations: *SW* salt wasting, *SV* simple virilizing, *GC* glucocorticoid dose, *SIDS* sudden infant death syndrome

Almost all deceased CAH children (*n* = 13) were besides their family doctor or paediatrician also under the care of a centre with a paediatric endocrinologist. Laboratory controls were performed regularly, and the three females classified as CAH-SV patients who were detected at birth by ambiguous genitalia had normal serum electrolytes and plasma renin activity (PRA) until the event that caused their death. The boy in the persistent vegetative state (case No. 8) was treated by the head physician of a county hospital. The centres rated the cooperation of the parents/patients as good in 9 cases and as poor in 5 cases. For 9 patients, information was provided on the date when they last presented as outpatients at the relevant centres. One patient presented as an outpatient and was released after being examined the day before he died (case No. 3), whereas the last outpatient examination of case No. 12 at a centre dated back to more than a year prior to his death. Half of the children died at home. Three children died in the hospital where they had also been treated as outpatients in the specialized outpatient department. Three children died immediately after admission to an external hospital. One child died of pneumonia in a home for severely handicapped children, where the boy (case No. 8) had been treated for apallic syndrome for 11 years. The event leading to the persistent vegetative state had occurred at the age of 1½ years. All children received treatment with hydrocortisone, with a mean dosage of 14.8 mg/m^2^ /day (range: 8.2–20 mg/m^2^ /day). Information on the fludrocortisone dosage was only provided in 9 cases, and the mean dosage was 163 μg/day (range: 100–375 μg/day).

Immediately before their deaths, 13 patients suffered from a high fever along with vomiting (*n* = 4), convulsions (n = 4), gastroenteritis with diarrhoea (*n* = 2), dyspnoea and/or upper respiratory tract infection (n = 2), and varicella (*n* = 1). One patient additionally suffered from a metabolic defect (MCAD defect). Considering the measures taken by the parents at home by themselves or upon consultation with the attending physicians, treatment of the children with elevated body temperature always focused on lowering the fever. In two cases, the parents also used antiemetic suppositories. The hydrocortisone dosage was doubled in 2 cases. No symptomatic or specific treatment was provided in 7 cases.

According to the assessments by the attending centres, the cause of death in these patients was a lack or inadequate adaptation of glucocorticoid dosage to the acute stress situation. Five patients developed hypoglycaemia and severe convulsions with cerebral oedema. In case No. 9, the centre did not find any correlation with CAH and specified “sudden infant death syndrome” as the cause of death. The cause of death certified on the death certificate by the reporting physicians was “death of natural causes” in 11 cases, “death of non-natural causes” in 2 cases, and “not clarified” in one case. An autopsy was only performed in 4 of the 14 cases of death. The pathologist/forensic medical expert found severe cerebral oedema in 3 cases. The autopsy did not yield any new findings in one case.

## Discussion

Deaths in patients with classic CAH in the context of an acute adrenal crisis (Addisonian crisis) are described in the literature. Mortality in CAH children is generally considered higher and assumed to be between 2 and 13% [17–19, 21].

Studies on mortality in CAH patients from Finland and the United Kingdom performed before the introduction of newborn screening for CAH report 7 deaths in 108 CAH patients in Finland [22] and 8 deaths (7 girls) in 333 patients (219 female) in the UK [21].

In the UK, the mortality rate was 3 times higher than that in the general population, and primarily toddlers between 1 and 4 years of age died. Our data strongly confirm the published results. In the UK, most affected children descended from Indian immigrant families [21], whereas in Germany, only one child had an immigrant background. Almost all deceased children in the UK had been under the care of specialized centres, and a similar situation was found in Germany.

In the UK, the high portion of deceased girls is explained by the high portion of girls in the cohort analysed. In Germany, the number of deceased girls was also slightly higher than that of boys, but the number of girls who died before newborn screening was almost equal to the number of girls who died after screening. It has to be kept in mind that half of the CAH children died after the introduction of newborn screening. In Japan, 12 deaths in CAH children were reported 12 years after the introduction of a newborn screening program [23]. Two of these children died during the newborn period at the ages of 13 and 23 days, whereas 10 children died under replacement therapy. In Hungary, 19 of 235 CAH patients died, including 17 with the SW-form of CAH (8 boys, 9 girls) and 2 boys with the SV-form of the disease. Sixteen of 17 CAH patients with the SW-form of the disease died within their first year of life. The mortality rate in CAH children with SW was 11.3% in the first year of life and thus significantly higher than the rate of 2.29% found in the Hungarian population [24].

It is known that in the presence of feverish infections and/or other acute stress situations, CAH children can be affected by an adrenal crisis at any stage of life. A study from the UK defines an adrenal crisis as an acute event that affects a patient’s health and requires i.v. glucocorticoid treatment and inpatient care. In this study, a survey was performed in 122 adults with CAH, and the medical records of the adult patients were analysed from the time of diagnosis until the date when they last presented as outpatients. The incidence of adrenal crises was highest in the first 10 years of life (70%), but crises were also observed in adults [15]. The data from Germany is highly consistent with these results.

In CAH patients, both adrenocortical and adrenomedullary dysfunction can result in severe hyponatremia, hyperkalemia and hypoglycemia due to impaired production of cortisol, aldosterone and epinephrine secretion [25–27]. Data extracted from a population-based prospective long-term follow-up study of 102 children with classic CAH detected in neonatal screening in Bavaria show that 22 children experienced a salt wasting adrenal crises (seven also with low blood glucose) and 16 children hypoglycemic episodes during the first 6 years of life [14].

Although the causes of death and/or the concomitant circumstances resulting in death have not been clarified in some of our cases, we assume that inadequate adaptation of the glucocorticoid dosage is likely in most cases. Glucocorticoid deficiency results in failure of the body’s compensation mechanisms. The most common form of shock in childhood is hypovolemic shock, which occurs due to constant fluid loss, e.g., within the scope of gastroenteritis. Cardiac output decreases before the onset of arterial hypotension. Therefore, therapy for shock in children must be started before the onset of hypotension [28]. CAH children seem to have a markedly elevated risk of acute adrenal crisis in the presence of acute gastroenteritis due to dehydration and/or lack of drug uptake during diarrhoea and/or vomiting. In our survey, two children died while suffering from gastroenteritis, including one case in which salmonella was detected.

Altogether, acute adrenal insufficiency is very rare in childhood. Most physicians only know the symptoms from textbooks, and only a few paediatricians have specific experience in the treatment of adrenal crisis. Well-known symptoms include hyponatremia, vomiting, diarrhoea, hypoglycaemia, dysrhythmia due to hyperkalaemia and increasing disorientation. In a recent study, it was noted that, in contrast to hyperkalaemia, hyponatremia is a constant laboratory finding in children with primary adrenal insufficiency [29].

It was also reported that the symptoms and the neuro-radiologic findings can include severe cerebral oedema [30]. In our survey, autopsies in three deceased CAH children provided evidence of cerebral oedema. The occurrence of cerebral oedema and/or water intoxication has been associated with CAH since the 1930s from animal experiments following adrenalectomy and/or in patients with Addison’s disease [31–33]. The secretion of antidiuretic hormone is increased in these situations, and the mere supply of volume without adequate adaptation of the glucocorticoid dosage can result in water intoxication [34, 35].

According to calculations performed in Japan, one in 25–80 affected CAH children dies in childhood [23]. The mortality is 1 in 12 CAH patients in Hungary [24], 1 in 15 CAH patients in Finland [22], and 1 in 24 CAH patients in the UK [21]. The data available in Germany do not allow calculating mortality rates.

Half of the deceased patients died at home. The hydrocortisone dosage was only doubled in two of the 14 cases. Therefore, delayed timing and/or lack of administration of life-saving emergency medication and/or inadequate dosage adaptation to the acute stress situation might have triggered the adrenal crisis and could be the underlying cause of almost all deaths.

The medical care of a child with CAH requires a close cooperation between all involved parties e.g. general practitioner, paediatrician, and parents or other caregiver as well as a confidence in the recommendations given by the paediatric endocrinologist. Already in 1977, it was recommended in the first German Textbook of Paediatric Endocrinology, to increase the hydrocortisone dose 3–5 times in all CAH children with febrile infections, surgery or after an accident [30]. The current recommendations in Germany correspond to the standards published by the Endocrine Society [31].

From 2005 on, there was no report of a deceased CAH patient in Germany. This result is very encouraging and shows the effectiveness of programs which have been conducted over the past years to educate and train parents, patients, caregivers and practitioners in realizing and managing adrenal crisis situations [36]. Emphasis should be placed on communication and training, even in case of emergency, and the knowledge of caregivers of affected children should be checked at regular intervals.

## Conclusions

There were some limitations to our study. The data were obtained by a survey and covered a period from 1973 to 2015. Our survey covered the whole of Germany, but it is not certain whether all cases of deceased CAH patients were reported. We speculate that most patients who died between 1973 and 2004 had an inadequate stress dosing of glucocorticoids. We assume that the quality of adrenal crisis management had improved over the past years since no deceased CAH children were reported from 2005 on.

## Additional file

Additional file 1: Questionnaire to evaluate the background of deceased CAH children. (DOCX 15 kb)

## Abbreviations

CAH: Congenital adrenal hyperplasia
SW: Salt wasting
