# Supplementary material for: Mortality in children with classic congenital adrenal hyperplasia and 21-hydroxylase deficiency (CAH) in Germany
Source: BMC Endocr Disord. 2018 Jun 8;18:37. doi: 10.1186/s12902-018-0263-1 (PMC5994009; doi:10.1186/s12902-018-0263-1)
Supplement: Supplementary file 1 — Questionnaire to evaluate the background of deceased CAH children. (DOCX 15 kb) [file 12902_2018_263_MOESM1_ESM.docx]

**Addional file 1**

Questionnaire - Evaluation of Deaths

Children with Classic CAH Due to 21-Hydroxylase deficiency (CAH)

Name/Address of the centre:

Birth Date of patient:

Sex:

Nationality:

Saltwasting form of CAH or simple virilizing form of CAH:

Used glucocorticoid:

- glucorticoid dosage (per m^2^ BSA):

Used mineralocorticoid

- dosage (µg/d):

Compliance/adherence before adrenal crisis (e.g. outpatient visits per year):

Quality of CAH therapy (e.g. metabolic control):

Year of death:

Place of death (e.g. hospital, at home):

Clinical symptoms of adrenal crisis before death:

Laboratory data:

Management of adrenal crisis:

- by the parents/caregivers
- by the practitioner/paediatrician
- by hospital

Cause of death:

Assessment of death:

Autopsy:
